# Overall Survival Associated with Real-World Treatment Sequences in Patients with CLL/SLL in the United States

**DOI:** 10.3390/cancers17152592

**Published:** 2025-08-07

**Authors:** Joanna M. Rhodes, Naleen Raj Bhandari, Manoj Khanal, Dan He, Sarang Abhyankar, John M. Pagel, Lisa M. Hess, Alan Z. Skarbnik

**Affiliations:** 1 Rutgers Cancer Institute of New Jersey, New Brunswick, NJ 08901, USA; 2 Eli Lilly and Company, Indianapolis, IN 46285, USApagel_john@lilly.com (J.M.P.);; 3 Syneos Health, Morrisville, NC 27560, USA; 4 Novant Health Cancer Institute, Charlotte, NC 28204, USA

**Keywords:** treatment sequencing, CLL/SLL, real-world data, time-dependent covariate, targeted therapy, line of therapy

## Abstract

**Background/Objectives:** This study compared overall survival (OS) associated with common real-world treatment sequences in patients with chronic lymphocytic leukemia/small lymphocytic lymphoma (CLL/SLL) in the United States. **Methods**: Utilizing the nationwide Flatiron Health electronic health record-derived de-identified database, adult CLL/SLL patients who initiated systemic therapy (JAN2016-NOV2023) and received at least two lines of therapy (LoTs) were analyzed. Treatment regimens were categorized based on drug class, and most frequent (n ≥ 50) sequences (first LoT followed by [→] second LoT) were compared. OS from initiation of the first LoT was compared using multivariable Cox proportional hazard models, and adjusted hazard ratios with 95% CIs were reported. **Results**: Among 2354 eligible patients, n = 1711 (73%) received the 16 most frequent treatment sequences. Sequencing chemoimmunotherapy (CIT) → CIT (HR: 2.29 [1.23–4.28]), anti-CD20 monoclonal antibody (anti-CD20mab) monotherapy → CIT (1.95 [1.03–3.69]), and covalent Bruton tyrosine kinase inhibitor (cBTKi) monotherapy → anti-CD20mab monotherapy (2.00 [1.07–3.74]) were associated with worse OS compared to patients treated with cBTKi monotherapy → B-cell lymphoma 2 inhibitors (BCL2i) + anti-CD20mab (reference). **Conclusions**: OS associated with other sequences were not significantly different from the reference sequence in adjusted analyses, suggesting a lack of evidence for the optimal standard of care for sequencing the first two LoTs in real-world settings. Future research should reassess sequencing outcomes as novel treatments become adopted into clinical practice.

## Simple Summary

Most patients with chronic lymphocytic leukemia (CLL) receive multiple lines of treatment (LoTs) over their disease course. However, the best approach of sequencing treatments remains unclear. This study used a large database of health records from 2016 to 2023 to compare the sixteen most common treatment sequences in real-world practice. It aimed to determine which sequences were associated with better overall survival (OS) among CLL patients who had received at least two LoTs. The study found that patients who received anti-CD20 monoclonal antibody (anti-CD20mab) monotherapy followed by chemoimmunotherapy (CIT), covalent Bruton tyrosine kinase inhibitor (cBTKi) monotherapy followed by anti-CD20mab, or CIT followed by CIT had worse OS compared to those who received cBTKi monotherapy followed by B-cell lymphoma 2 inhibitors plus anti-CD20mab (reference). However, many other sequences showed no difference in OS versus the reference sequence. More research is needed as new treatments are adopted into clinical practice.

## 1. Introduction

The management of chronic lymphocytic leukemia and small lymphocytic lymphoma (CLL/SLL; hereafter referred to as CLL) has evolved significantly over the last decade, from using chemotherapy-based treatments to targeted therapies, primarily in earlier lines of treatment [1]. With advancements in clinical development and availability of several novel classes of targeted therapies, current treatment recommendations have evolved to include covalent Bruton tyrosine kinase inhibitor (cBTKi)- and/or B-cell lymphoma 2 inhibitor (BCL2i)-based regimens in earlier lines of treatment, with or without an anti-CD20 monoclonal antibody (anti-CD20mab) [1,2]. Other therapies (e.g., phosphoinositide 3-kinase inhibitors; PI3Ki, non-covalent BTKi; ncBTKi, chimeric antigen receptor cell therapy; CAR-T) are also available as additional treatment options [3,4,5]. Chemotherapy-based treatment options are also available (e.g., regimens based on bendamustine, fludarabine, or cyclophosphamide); however, their use in earlier lines of treatment is less preferred, as reflected in the latest recommendations from the Lymphoma Research Foundation [6]. The choice of treatment in part depends on the presence/absence of mutations in prognostic biomarkers and/or other disease characteristics, potentially resulting in various treatment sequencing strategies and outcomes [7,8]. Additionally, the evaluation of key biomarkers, including deletion at chromosome 17p13.1 [del(17p)] and mutations in the *TP53* gene and/or *IGHV*, are particularly recommended when considering chemoimmunotherapy (CIT; chemotherapy plus anti-CD20mab) and ibrutinib [9,10,11].

Despite significant advances in therapeutic options, CLL remains largely incurable, frequently necessitating multiple lines of treatment over the disease course. The optimal sequencing of available treatments is unclear, often relying on anecdotal evidence and clinician experience, leading to variability in clinical practice [12,13]. The variability of utilization of available treatment options underscores the need for additional research to determine the most effective sequences of treatment to optimize patient outcomes. When evaluating treatment sequences in oncology, overall survival (OS) becomes a critical endpoint because it directly measures the intended goal of therapy—improving survival so that patients with CLL have similar long-term outcomes as those without. OS may provide a comprehensive assessment of the cumulative effects of different therapies over time. Unlike surrogate endpoints, OS encompasses all potential benefits and harms of a sequence of treatments, including long-term side effects and quality of life impacts [14]; however, necessary adjustments in statistical analysis must be made to ensure it is not confounded by additional treatments beyond the sequence evaluated in a study.

No difference in unadjusted OS was reported in a UK-based retrospective study among patients with CLL who received first-line CIT vs. targeted therapy (median [95% CI] years: 8.71 [7.80–9.97] vs. 8.37 [6.63–NE]) between 2005 and 2020 [15]. The study only evaluated first-line treatment, without adjusting for the potential effects of treatments received beyond first-line and differences in patient and clinical characteristics. There is a need for more comprehensive sequencing studies with robust methods that account for time-related confounding. Addressing the effect of potential confounding factors over time that could obscure outcomes (like OS) associated with treatment sequences is crucial in this type of research. One way to address this is by using time-dependent covariate modeling, which allows us to adjust for the impact of factors that change over time on observed outcomes [16,17]. This observational comparative effectiveness research study compared OS associated with frequently observed real-world treatment sequences in patients with CLL treated in the first- and second-line setting in the U.S. using multivariable modeling including time-dependent covariates.

## 2. Materials and Methods

### 2.1. Study Design and Data Source

**Figure 1:**
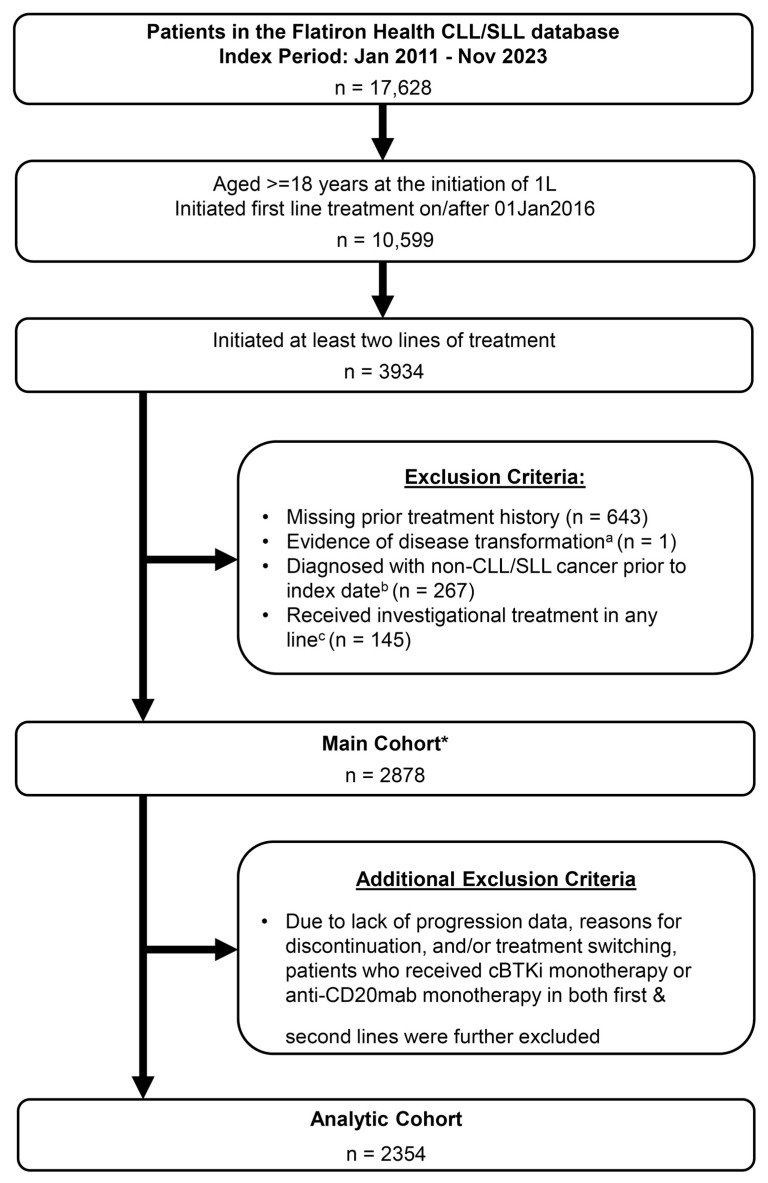
CONSORT diagram of the study population. * After review of treatment sequences in the Main Cohort, additional exclusion criteria were applied. ^a^ Transformation date indicates whether the patient’s disease transformed to a more aggressive hematological malignancy including Richter’s transformation (DLBCL), Hodgkin’s Lymphoma (HL), and Prolymphocytic leukemia (PLL) within first five LoT. ^b^ Defined as having non-CLL/SLL cancer diagnosis if they have two or more primary non-CLL/SLL cancer diagnosis codes at least 30 days apart. ^c^ Clinical Study Drug either alone or in any form of combination.

This retrospective cohort study included patients with CLL from the nationwide Flatiron Health electronic health record-derived de-identified database (Figure 1). The Flatiron Health database is a U.S. nationwide longitudinal database, comprising de-identified patient-level structured (e.g., laboratory values, prescribed drugs) and unstructured data (e.g., physician’s notes, biomarker reports), curated via technology-enabled abstraction. The database includes patient demographics, treatment, and clinical outcomes from a diverse pool of data [18]. During the study period, the de-identified data originated from approximately 280 cancer clinics in the U.S. (~800 unique sites of care) [19,20]. A few additional rules were applied to the available LoT table in the database based on clinical judgment by the study team to address some nuanced discrepancies observed in the study cohort (Supplementary Table S1).

The data from January 2011 to November 2023 were used, with the index date defined as the initiation date of the first line of therapy that occurred on or after January 2016. The follow-up period was the time from the index date to the latest of either the last activity date or date of death (based on the last recorded observation in the drug episode, visit, and telemedicine tables).

### 2.2. Study Population

Patients with CLL/SLL aged ≥18 years who initiated first-line therapy on/after 1 January 2016 and who received at least two LoTs were included. Patients with evidence of unknown prior treatment history, disease transformation within first five LoTs, diagnosis of other non-CLL cancers prior to initiation of the first line of therapy, or receipt of investigational treatment in any line of therapy were excluded (Figure 1). Applying these criteria resulted in the main study cohort (Main Cohort). Due to the lack of data about progression and/or other reasons for discontinuation and treatment switching, patients who received cBTKi monotherapy or anti-CD20mab monotherapy in both the first and second line of therapy were excluded as an additional post hoc exclusion. This criterion was applied because it was unclear if the same agent was used in a second line, or if the drug was simply a continuation of first-line therapy. To avoid this potential bias in determining sequences, the analytic study cohort (Analytic Cohort) was used for analyses after this additional exclusion criterion was applied.

### 2.3. Outcome

OS was defined as the duration (months) from the initiation of the first line of therapy to death. Patients without a death event were censored at their last activity date.

### 2.4. Statistical Analyses

Treatment regimens were grouped into pre-defined, mutually exclusive categories based on drug class (Supplementary Table S2). Patients were grouped into unique categories based on the sequence of treatments received in the first and second lines of therapy (first line followed by [→] second line). Baseline patient characteristics of the Analytic Cohort and those who received the most common (n ≥ 50) unique groups of sequences were described using descriptive statistics.

Sequences with at least 50 patients were compared using Kaplan–Meier (KM) and multivariable analyses. The unadjusted median (95% CI) OS and 4-year landmark rates were estimated using the KM method and compared using a log-rank test. Two separate multivariable Cox proportional hazard models (two-sided significance level of 0.05) were used to make adjusted comparisons of OS between sequences with at least 50 patients. Model 1 (M1) adjusted for baseline (at/prior to index date) factors including age at index, sex, race/ethnicity, socioeconomic status, practice type, disease subtype, Rai stage, ECOG performance status (PS) at index, del(11q), del(13q), trisomy 12 (+12), del(17p)/*TP53* mutation, *IGHV* mutation status, index year, and time since initial diagnosis to index. Model 2 (M2) additionally adjusted for cumulative number of lines of therapys received along with their duration as a blended time-dependent covariate to account for potential differences in OS due to treatments received beyond the first two lines of therapy. The adjusted hazard ratios (aHR) and 95% CIs were reported for each model using cBTKi monotherapy → BCL2i + anti-CD20mab as the reference sequence, as it is perceived to be one of the more commonly used sequences in the current clinical practice. All analyses were conducted on both the Main and Analytic Cohort, using SAS v9.4 (SAS Institute Inc., Cary, NC, USA).

Post hoc subgroup analysis: Given the scarcity of evidence on this topic in elderly patients and among various racial/ethnic groups, a descriptive post hoc subgroup analysis was conducted to describe the most frequent treatment sequences observed and their associated unadjusted OS using the KM method by age (≤75 years and >75 years at the initiation of first-line treatment) as well as by race/ethnicity (non-Hispanic White, non-Hispanic Black, and Hispanic).

Unmeasured confounding is a common limitation in observational research. In this study, e-value was estimated to assess the impact of unmeasured (or unobserved) confounding on the estimated treatment effect. E-value represented the minimum strength of association that a hypothetical unmeasured confounder would need to have with both the ‘groups being compared’ (i.e., sequences of treatment) and the outcome of interest (i.e., OS) on the risk ratio scale, conditional on the measured covariates, to nullify a specific estimated treatment effect [21]. A larger e-value indicates that a greater amount of unmeasured confounding is required to negate the estimated treatment effect. The e-value for the estimated a HR associated with each treatment sequence in M1 and M2 was calculated using SAS v9.4 according to the formula published by Linden and Mathur, 2020 [22] and were comfirmed using evalues.HR function in R v4.1.0, from the EValue package.

## 3. Results

Overall, 2878 patients with CLL met the eligibility criteria and were included in the Main Cohort, while 2354 patients comprised the Analytic Cohort after implementing the additional exclusion criterion (Figure 1). Of the 2354 patients in the Analytic Cohort, 73% (1711/2354) of patients received the most frequent sequences of treatment, where each sequence category had a minimum of 50 patients; this subset of 73% patients was included in the comparative analyses.

### 3.1. Baseline Patient Characteristics

Patients in the Analytic Cohort (N = 2354) had a median (interquartile range, IQR) time from initial diagnosis of CLL to the initiation of the first line of treatment (index date) of 23 (3–60) months and a median follow-up of 45 (25–67) months from the index date. The median age at the index date was 71 (64–78) years; 64% were male, 63% were non-Hispanic White, and 87% received treatment in the community setting. Among patients with available data, 12% (217/1848) had del(17p)/*TP53* mutation, 62% (545/884) had unmutated *IGHV*, 92% (1534/1670) had ECOG PS 0/1, and 64% (904/1410) were initially diagnosed with Rai stage 0/I. More than one-third of patients received treatments beyond the first two lines (35%). Extended patient demographic and clinical characteristics are listed in Table 1. These baseline patient characteristics of the Analytic Cohort were similar to those of its subset of patients included in comparative analyses (n = 1711), with similar median follow-up (47 [26–69] months). Corresponding baseline characteristics and outcomes for the Main Cohort are provided in the supplementary content (Supplementary Table S3).

### 3.2. Frequently Observed Treatment Sequences

**Figure 2:**
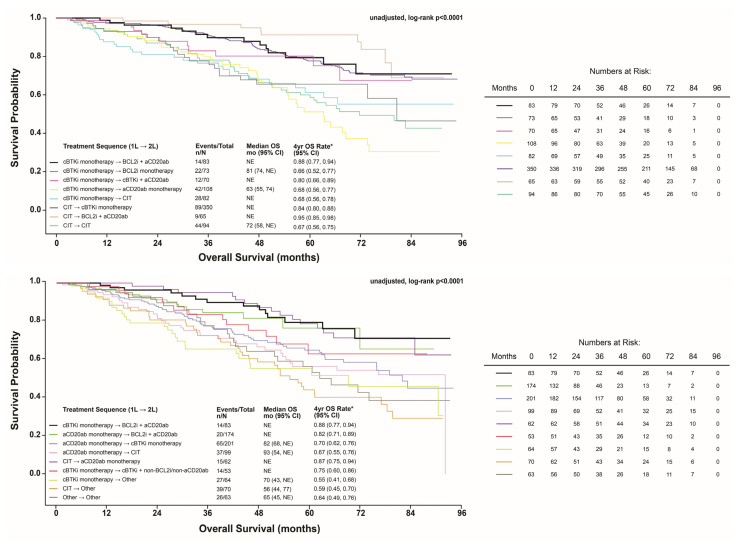
Kaplan–Meier plot of most frequently observed treatment sequences in the Analytic Cohort. * Point estimate for OS at 4 years (48 months) based on survival distribution function. Other included any treatment regimen that was not classified as cBTKi monotherapy, non-cBTKi monotherapy, aCD20abmab monotherapy, BCL2i monotherapy, PI3Ki monotherapy, BCL2i + aCD20abmab only, chemotherapy only, chemoimmunotherapy (aCD20mab + chemotherapy only), BCL2i + cBTKi only, BCL2i + ncBTKi only, cBTKi + aCD20mab only, ncBTKi + aCD20mab only, BCL2i + cBKTi + aCD20mab only, BCL2i + ncBKTi + aCD20mab only, PI3Ki + chemotherapy only, BCL2i + non-BTKi/non-aCD20mab, cBTKi + non-BCL2i/non-aCD20mab, and ncBTKi + non-BCL2i/non-aCD20mab. CIT; chemoimmunotherapy (anti-CD20 monoclonal antibody + chemotherapy). Note: KM plot was split for visualization purposes only.

**Figure 3:**
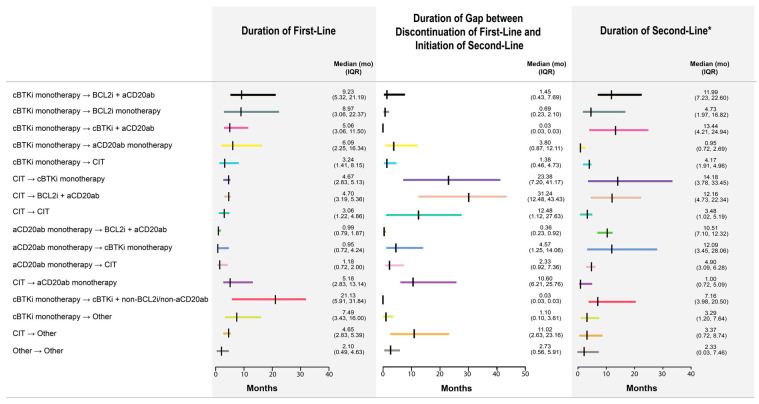
Durations of line and gap between first two lines within each treatment sequence in the Analytic Cohort. * These durations do not account for censoring as not all patients received a subsequent line of treatment and are descriptive only. Vertical bars represent the median duration of each line or gap between two lines within a sequence. Horizontal bars represent interquartile ranges around the median duration of each line or gap between two lines within a sequence.

Of the 2354 patients in the Analytic Cohort, 73% (n = 1711) received the 16 most frequent (n ≥ 50) treatment sequences (Figure 2; Main Cohort can be viewed in Supplementary Figure S1). These sequences included (i) CIT → cBTKi monotherapy (n = 350; 20.5%); (ii) anti-CD20mab monotherapy → cBTKi monotherapy (n = 201; 11.7%); (iii) anti-CD20mab monotherapy → BCL2i + anti-CD20mab (n = 174; 10.2%); (iv) cBTKi monotherapy → anti-CD20mab monotherapy (n = 108; 6.3%); (v) anti-CD20mab monotherapy → CIT (n = 99; 5.8%); (vi) CIT → CIT (n = 94; 5.5%); (vii) cBTKi monotherapy → BCL2i + anti-CD20mab (n = 83; 4.9%); (viii) cBTKi monotherapy → CIT (n = 82; 4.8%); (ix) cBTKi monotherapy → BCL2i monotherapy (n = 73; 4.3%); (x) cBTKi monotherapy → cBTKi + anti-CD20mab (n = 70; 4.1%); (xi) CIT → other (n = 70; 4.1%); (xii) CIT → BCL2i + anti-CD20mab (n = 65; 3.8%); (xiii) cBTKi monotherapy → other (n = 64, 3.7%); (xiv) other → other (n = 63, 3.7%); (xv) CIT → anti-CD20mab monotherapy (n = 62; 3.6%); and (xvi) cBTKi monotherapy → cBTKi + non-BCL2i/non-anti-CD20mab (n = 53; 3.1%). For each sequence, the median (IQR) duration of first-line therapy, duration of the gap between the discontinuation of first-line therapy and initiation of second-line therapy, and duration of second-line therapy (unaccounted for censoring) are graphically depicted in Figure 3 (Main Cohort can be viewed in Supplementary Figure S2).

### 3.3. Unadjusted Comparison of OS

Median (95% CI) OS was not estimable for most sequences except for patients who received (i) cBTKi monotherapy → anti-CD20mab monotherapy: 63 (55–74) months; (ii) cBTKi monotherapy → BCL2i monotherapy: 81 (74–NE) months; (iii) CIT → CIT: 72 (58–NE) months; (iv) anti-CD20mab monotherapy → cBTKi monotherapy: 82 (68–NE) months; and (v) anti-CD20mab monotherapy → CIT: 93 (54–NE) months. The estimated median OS and 4-year OS rate for each treatment sequence are shown in Figure 2.

### 3.4. Adjusted Comparison of OS

**Figure 4:**
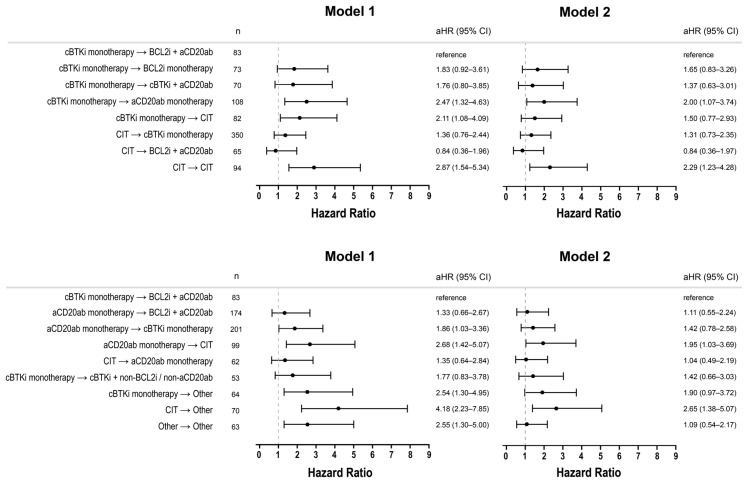
Adjusted hazard ratios from multivariable Cox proportional hazard models for comparison of OS across most frequently observed treatment sequences in the Analytic Cohort. Model 1 adjusted for baseline (at/prior to index date) factors including age at index, sex, race/ethnicity, socioeconomic status, practice type, disease subtype, Rai stage, ECOG at index, d11q, d13q, tri12, del17p/*TP53*, *IGHV*, index year, and time since initial diagnosis to index. Model 2 additionally adjusted for cumulative number of lines of therapys received along with their duration as a blended time-dependent covariate to account for potential differences in OS due to treatments received beyond the first two lines of therapy. Reference sequence: cBTKi monotherapy → BCL2i + anti-CD20 monoclonal antibody. CIT; chemoimmunotherapy (anti-CD20 monoclonal antibody + chemotherapy). Note: Forest plot was split for visualization purposes only.

Compared to patients who received cBTKi monotherapy → BCL2i + anti-CD20mab (reference sequence), treatment with the following sequences was associated with statistically significantly (*p* < 0.05) worse OS (aHR [95% CI]) in both M1 and M2: (i) cBTKi monotherapy → anti-CD20mab monotherapy (M1: 2.47 [1.32–4.63], M2: 2.00 [1.07–3.74]); (ii) anti-CD20mab monotherapy → CIT (M1: 2.68 [1.42–5.07], M2: 1.95 [1.03–3.69]); (iii) CIT → CIT (M1: 2.87 [1.54–5.34], M2: 2.29 [1.23–4.28]); and (iv) CIT → Other (M1: 4.18 [2.23–7.85], M2: 2.65 [1.38–5.07]) (Figure 4).

Treatment sequences that did not differ statistically significantly versus the reference sequence in both M1 and M2 included (v) CIT → cBTKi monotherapy (M1: 1.36 [0.76–2.44], M2: 1.31 [0.73–2.35]); (vi) anti-CD20mab monotherapy → BCL2i + anti-CD20mab (M1: 1.33 [0.66–2.67], M2: 1.11 [0.55–2.24]); (vii) cBTKi monotherapy → BCL2i monotherapy (M1: 1.83 [0.92–3.61], M2: 1.65 [0.83–3.26]); (viii) cBTKi monotherapy → cBTKi + anti-CD20mab (M1: 1.76 [0.80–3.85], M2: 1.37 [0.63–3.01]); (ix) CIT → BCL2i + anti-CD20mab (M1: 0.84 [0.36–1.96], M2: 0.84 [0.36–1.97]); (x) CIT → anti-CD20mab monotherapy (M1: 1.35 [0.64–2.84], M2: 1.04 [0.49–2.19]); and (xi) cBTKi monotherapy → cBTKi + non-BCL2i/non-anti-CD20mab (M1: 1.77 [0.83–3.78], M2: 1.42 [0.66–3.03]). All remaining treatment sequences were significantly different versus the reference sequence in M1, but were not significantly different in M2: (xii) CIT → cBTKi monotherapy (M1: 1.36 [0.76–2.44], M2: 1.31 [0.73–2.35]); (xiii) cBTKi monotherapy → CIT (M1: 2.11 [1.08–4.09]), M2: 1.50 [0.77–2.93]); (xiv) cBTKi monotherapy → Other (M1: 2.54 [1.30–4.95], M2: 1.90 [0.97–3.72]); and (xv) anti-CD20mab monotherapy → cBTKi monotherapy (M1: 1.86 [1.03–3.36], M2: 1.42 [0.78–2.58]).

These findings were similar when evaluated among the most frequently observed sequences from the Main Cohort (Supplementary Figure S3). Additionally, OS was not statistically significantly different in patients who received treatment with cBTKi monotherapy → cBTKi monotherapy (M1: 0.77 [0.42, 1.40], M2: 0.72 [0.40, 1.33]) compared to the reference sequence; however, the interpretation is limited due to the lack of data on progression and/or reason for treatment discontinuation/treatment switching.

### 3.5. Post Hoc Subgroups

The frequency of each treatment sequence along with the unadjusted 4-year OS probability estimates for each subgroup are presented in Table 2.

### 3.6. Outcomes by Age

Of the n = 1711 patients from the Analytic Cohort, 66% were ≤75 years old while 34% were >75 years old at the initiation of first-line treatment. The frequency distribution of most treatment sequences was different between both groups. Younger patients (≤75 years) more frequently received CIT → cBTKi monotherapy (24%), anti-CD20mab monotherapy → BCL2i + anti-CD20mab (11%), and anti-CD20mab monotherapy → cBTKi monotherapy (9%), while older patients (>75 years) more frequently received anti-CD20mab monotherapy → cBTKi monotherapy (17%), CIT → cBTKi monotherapy (13%), and cBTKi monotherapy → anti-CD20mab monotherapy (9%). The unadjusted, estimated 4-year OS rates ranged from 64 to 96% in younger patients, whereas they ranged from 35 to 92% in older patients across various sequences. The highest 4-year OS rate in both groups was associated with CIT → BCL2i + anti-CD20mab (younger: 96% and older: 92%), although only a small proportion of patients in both groups received that sequence (younger: 5%; older: 2%).

### 3.7. Outcomes by Race/Ethnicity

Of the n = 1711 patients in the Analytic Cohort, 63% were Non-Hispanic White (NHW), 7% were Non-Hispanic Black (NHB), and 4% were Hispanic (H). The frequency distribution of most treatment sequences was different between the three racial ethnic groups. Patients were more likely to receive following sequences with variable proportions across three groups: CIT → cBTKi monotherapy (NHW: 21%; NHB: 31%; H: 21%), anti-CD20mab monotherapy → cBTKi monotherapy (NHW: 12%; NHB: 6%; H: 13%), and anti-CD20mab monotherapy → BCL2i + anti-CD20mab (NHW: 10%; NHB: 7%; H: 17%). The unadjusted, estimated 4-year OS rates ranged from 58 to 98% (NHW), 25–100% (NHB), and 67–100% (H) across various sequences within the three groups.

The potential impact of unmeasured confounding was assessed by estimating the e-value associated with each treatment sequence and its hazard ratio. The e-values ranged between 1.5 and 4.7 for various treatment sequences in M1, whereas they ranged between 1.2 and 3.3 in M2 (Supplementary Table S4), indicating that moderate-to-substantial unmeasured confounding would be needed to nullify the treatment effects observed in this analysis depending on the treatment sequence compared with the reference.

## 4. Discussion

The treatment landscape in CLL has significantly evolved; however, the optimal strategy for sequencing treatments remains unknown. With a median follow-up of 47 months, this real-world study compared OS between the sixteen most frequent treatment sequences based on first and second lines of treatment received in the U.S. Among the very few sequences with an estimable median, the lowest mOS was observed in patients who received CIT → other (56 months) and cBTKi monotherapy → anti-CD20mab monotherapy (63 months). Additionally, sequential regimens including CIT → CIT, anti-CD20mab monotherapy → CIT, and cBTKi monotherapy → anti-CD20mab monotherapy were associated with poorer OS outcomes compared to those who received cBTKi monotherapy → BCL2i + anti-CD20mab (reference) after adjusting for baseline and time-dependent covariates. The OS outcomes associated with other sequences were not significantly different from the reference sequence in adjusted analyses, indicating a lack of evidence for the optimal standard of care for sequencing the first two lines of treatment in real-world settings. These findings also highlight the complexity of treatment selection and sequencing, where no clear treatment sequence associated with survival benefit is observed.

Despite limiting the cohort to those who started first-line treatment in 2016 or later, most of the frequently observed sequences did not include targeted therapies within the first two lines of treatment (75%, 12/16). This may reflect the slower integration of newer treatment modalities over time, primarily in the community setting. As novel agents are approved and incorporated into routine clinical practice, there is an opportunity to investigate new sequences, such as continuing treatment with BTK pathway inhibition (e.g., cBTKi monotherapy → ncBTKi or cBTKi monotherapy). Given the promising results from recent studies, such as the BRUIN CLL-321 study in the post-cBTKi setting [25], there is potential for these novel agents to improve patient outcomes.

There is limited published evidence regarding treatment sequencing in CLL and OS outcomes. Robak et al. [26], in their integrated analysis of data from the clinical RESONATE-2trial and a real-world PHEDRA study, reported superior OS outcomes in patients who received first-line ibrutinib → either any treatment or no treatment in the second line, compared to those who received CT (chemotherapy) → ibrutinib (5-year OS rates: 84% vs. 62%) or CT/CIT → ibrutinib (5-year OS rates based on data from RESONATE-2 and PHEDRA: 87% vs. 63%; PHEDRA alone: 89% vs. 82%). However, more than 80% of patients who received ibrutinib as first-line therapy did not receive any subsequent treatment in the second line, which may have impacted the reported outcomes despite adjustments in their study. Similarly, statistically indifferent 4-year OS rates were reported by Ghosh et al. [27] using the informCLL registry among patients who received first-line ibrutinib (83%) or CIT (82%). However, the proportion of patients who received second-line treatment and the type of treatment received were not reported. This limits additional comparison of findings observed in the current study where each sequence was defined based on treatments received in the first and second lines. Understanding and adjusting for subsequent lines of treatment is essential because of their potential to impact outcomes, as observed in this study where the 4-year OS rate ranged between 55% and 88%, depending on the treatment received in the second line after receiving first-line cBTKi monotherapy. Additionally, the 4-year OS rate was 84% for patients who received CIT → cBTKi monotherapy in this study. In contrast, the 5-year OS rate among patients who received CT/CIT → ibrutinib ranged between 62% and 82%, depending on the datasets used, as reported in the study by Roback et al. [26].

Using data from Optum claims, Huntington et al. [28] reported that the two most frequently observed targeted therapy-based sequences in patients who received at least two LoTs were BTKi → BTKi (66–80%) and BTKi → BCL2i (18–26%). Similarly, Roeker et al. [29], reported higher proportions of patients receiving cBTKi → cBTKi and cBTKi → BCL2i using ConcertAI data. The distributions of these sequences are comparable to those in the current study, when the most frequent sequences in Analytic and Main Cohorts are restricted to those containing targeted therapies within both lines. However, the applicability of findings from these two studies are limited as they did not report OS outcomes associated with these sequences of treatments [28,29], which is an important factor in clinical decision-making. Furthermore, many other published studies have described treatment patterns and outcomes, primarily focusing on response rate, progression-free survival, and time-to-next treatment, categorized by specific LoTs [30,31,32,33,34,35]. However, none of these have reported OS outcomes by the specific sequence of treatments received in first and second lines, along with outcomes measured from the initiation of the first line. The current study, therefore, builds on the existing literature and addresses their limitations by comparing OS outcomes across the most frequently observed specific sequences of treatments within first two lines using a robust methodology.

Given the scarcity of evidence in elderly patients and racial/ethnic subgroups, this study also described the frequency of treatment sequences received and their associated unadjusted 4-year survival rates. For instance, younger patients, from the initiation of first-line treatment (≤75 years), were more likely to receive CIT → cBTKi monotherapy (24%) compared to older patients (>75 years; 13%). Additionally, younger patients were more likely to have a better survival rate at 4 years from the initiation of first-line treatment (younger: 86%; older: 77%) in the unadjusted analysis in this study. Similarly, Non-Hispanic Black patients (31%) were more likely to receive CIT → cBTKi monotherapy compared to Hispanic (21%) and Non-Hispanic White (21%) patients. However, their unadjusted 4-year OS rates (NHB: 86%; H: 86%; NHW: 84%) associated with this sequential treatment were relatively similar across all three groups described in this study. These findings suggest that the types of treatment sequences received and unadjusted outcomes may or may not vary by these subgroups and should be further evaluated in future studies, particularly considering adjusted analyses. A recent study in patients with CLL in the U.S. reported no difference in OS (measured from the first line of treatment) by racial/ethnic characteristics in both unadjusted and adjusted analyses [36]. However, inherent differences in study design and analyses conducted between the two studies, such as the evaluation of outcomes by the sequence of first and second lines of therapies in the present study versus the evaluation of outcomes in a cohort of patients regardless of the type of first-line treatment received across racial/ethnic subgroups, limit the comparability of findings. Additionally, it remains unknown and could be explored in future research whether findings in these subgroups reported in this study will hold after adjusting for differences in baseline characteristics, which have been previously reported to be different [37,38,39]. Nevertheless, these descriptive findings underscore the importance of addressing inequities in caring for patients with CLL and ensuring that all patients have access to the most effective treatments.

One of the key strengths of this study is the implementation of complex comparisons adjusted for baseline covariates and, more importantly, accounting for key post-baseline factors as time-dependent covariates that may impact OS. While the approach of incorporating the cumulative number of lines of treatment, including its duration, as a blended time-dependent covariate (in M2) is not traditionally applied, it aids in conducting a more comprehensive evaluation of treatment outcomes over extended periods. Notably, introducing this time-dependent covariate in M2 resulted in (i) a reduction in the magnitude of effect for almost all treatment sequences observed in M1 and (ii) non-significant hazard ratios that were statistically significant in M1, demonstrating the importance of taking this approach when evaluating the impact of treatment sequencing on OS. By including a blended time-dependent covariate, this study accounted for the potential effect that subsequent lines of therapy could have on OS. One of the significant limitations of this retrospective analysis was the exclusion of all infrequent sequences (n < 50), including BCL2i + anti-CD20mab → cBTKi monotherapy (n = 12), another emerging standard of care sequence in CLL. No adjustments were made for multiple comparisons in the multivariable analyses. Moreover, the potential for unobserved confounding cannot be excluded due to a lack of data such as the intended duration of a treatment regimen (fixed or continuous), reasons for discontinuation and/or treatment switching, and geographic region. However, the e-value associated with each treatment sequence and its hazard ratio were estimated to assess the potential impact of unmeasured confounding in this study [21]. The e-values ranged between 1.5 and 4.7 for various treatment sequences in M1 and between 1.2 and 3.3 in M2 (Supplementary Table S4), indicating that moderate-to-substantial unmeasured confounding would be needed to nullify the treatment effects observed in this analysis depending on the treatment sequence compared with the reference. The study design may introduce selection bias, as patients who only received first-line treatment during the study period or died before initiating second-line treatment were excluded. However, the impact of this potential bias on the observed findings could be in either direction, which remains unknown. Lastly, the treatment sequences in this study were defined based on pre-specified drug classes (Supplementary Table S2); however, outcomes may vary depending on the specific composition of treatment regimens within each drug class category. This aspect could be explored in future studies with larger sample sizes.

## 5. Conclusions

In a cohort of CLL patients treated mainly in community-based settings in the U.S., sixteen common treatment sequences were compared. Sequential use of cBTKi monotherapy followed by BCL2i + anti-CD20mab (reference) showed significantly improved OS compared to some CIT- or anti-CD20mab-based sequences. However, OS outcomes of other sequences were not significantly different from the reference sequence, suggesting that there is no clear optimal standard of care for sequencing the first two lines of treatment in this real-world population. Future research should be conducted to reassess sequencing outcomes as novel monotherapy and/or combination treatments are adopted into routine clinical practice.

## Figures and Tables

**Table 1:** Baseline patient characteristics of the Analytic Cohort.

Characteristics—Analytic Cohort	Overall N = 2354	Patients Included in Comparative Analyses N = 1711 ^a^
**Age, median (IQR)**	71 (64, 78)	71 (63, 78)
**Age subgroups, n (%)**		
≤75 years	1547 (65.7)	1129 (66.0)
>75 years	807 (34.3)	582 (34.0)
**Sex, n (%)**		
Male	1496 (63.6)	1083 (63.3)
Female	858 (36.4)	628 (36.7)
**Combined Ethnicity and Race, n (%)**		
Non-Hispanic White	1489 (63.3)	1078 (63.0)
Non-Hispanic Black/African American	163 (6.9)	126 (7.4)
Hispanic	107 (4.5)	72 (4.2)
Other ^b^	595 (25.2)	435 (25.4)
**Socioeconomic status ^c^, n (%)**		
1	290 (12.3)	216 (12.6)
2	358 (15.2)	259 (15.1)
3	444 (18.9)	325 (19.0)
4	554 (23.5)	417 (24.4)
5	532 (22.6)	371 (21.7)
Missing	176 (7.5)	123 (7.2)
**Practice type, n (%)**		
Academic	308 (13.1)	215 (12.6)
Community	2046 (86.9)	1496 (87.4)
**Disease subtype, n (%)**		
CLL	2153 (91.5)	1555 (90.9)
SLL	201 (8.5)	156 (9.1)
**Year of initiation of first LoT, n (%)**		
2016	491 (20.9)	384 (22.4)
2017	418 (17.8)	339 (19.8)
2018	368 (15.6)	274 (16.0)
2019	327 (13.9)	220 (12.9)
2020	274 (11.6)	181 (10.6)
2021	248 (10.5)	154 (9.0)
2022	164 (7.0)	111 (6.5)
2023	64 (2.7)	48 (2.8)
**Total Number of LoT received, n (%)**		
2	1539 (65.4)	1153 (67.4)
3	512 (21.8)	353 (20.6)
4+	303 (12.9)	205 (12.0)
**Patients with data available, n (%) ^d^**		
**ECOG PS**	**n = 1670**	**n =** **1224**
0–1	1534 (91.9)	1119 (91.4)
2–4	136 (8.1)	105 (8.6)
**Rai Stage**	**n = 1410**	**n = 1039**
0–I	904 (64.1)	651 (62.7)
II–IV	506 (35.9)	388 (37.3)
**IGHV**	**n = 884**	**n = 653**
Mutated	339 (38.3)	247 (37.8)
Unmutated	545 (61.7)	406 (62.2)
**del(11q)**	**n = 1836**	**n = 1355**
No	1512 (82.4)	1122 (82.8)
Yes	324 (17.6)	233 (17.2)
**del(13q)**	**n = 1852**	**n = 1371**
No	1015 (54.8)	749 (54.6)
Yes	837 (45.2)	622 (45.4)
**del(17p)/TP53**	**n = 1848**	**n = 1365**
No	1631 (88.3)	1217 (89.2)
Yes	217 (11.7)	148 (10.8)
**Trisomy 12**	**n = 1829**	**n = 1355**
No	1342 (73.4)	995 (73.4)
Yes	487 (26.6)	360 (26.6)

^a^ Included patients who received the most common treatment sequences (n ≥ 50). ^b^ Other includes 22 (0.9%) Asian, 70 (3.0%) other, and 503 (21.4%) missing/unknown in the overall population; and 16 (0.9%) Asian, 54 (3.2%) other, and 365 (21.3%) missing/unknown in the patients included in comparative analysis population. ^c^ Socioeconomic status; an area-level indicator of patients’ socioeconomic status (1-low; 5-high), which was calculated according to the Yost Index (incorporating income, home values, rental costs, poverty, blue-collar employment, unemployment, and education information) using Census block group (i.e., neighborhood) data from the American Community Survey (2015–2019) [23,24]. ^d^ Excludes patients with missing data.

**Table 2:** Post hoc Description of Frequent Treatment Sequences and Associated Unadjusted Overall Survival by Subgroups in the Analytic Cohort.

Frequently Observed Treatment Sequence (n = 1711)	n (%) Estimated 4-Year Rate (95% CI) of OS				
	Age at Initiation of First-Line Treatment, Years		Race/Ethnicity *		
	≤75	>75	Non-Hispanic White	Non-Hispanic Black	Hispanic
	1129/1711 (66%)	582/1711 (34%)	1078/1711 (63%)	126/1711 (7%)	72/1711 (4%)
CIT → cBTKi monotherapy	274 (24.3%) 0.86 (0.81, 0.90)	76 (13.1%) 0.77 (0.66, 0.86)	228 (21.2%) 0.84 (0.79, 0.89)	39 (31.0%) 0.86 (0.69, 0.94)	15 (20.8%) 0.86 (0.54, 0.96)
Anti-CD20mab monotherapy → cBTKi monotherapy	102 (9%) 0.88 (0.78, 0.93)	99 (17.0%) 0.51 (0.39, 0.62)	127 (11.8%) 0.72 (0.62, 0.80)	8 (6.3%) 0.45 (0.11, 0.75)	9 (12.5%) 0.86 (0.33, 0.98)
Anti-CD20mab monotherapy → BCL2i + Anti-CD20mab only	129 (11.4%) 0.85 (0.69, 0.93)	45 (7.7%) 0.71 (0.50, 0.84)	103 (9.6%) 0.78 (0.63, 0.87)	9 (7.1%) 1.00 (1.00, 1.00)	12 (16.7%) NE (NE, NE)
cBTKi monotherapy → Anti-CD20mab monotherapy	55 (4.9%) 0.86 (0.71, 0.94)	53 (9.1%) 0.47 (0.30, 0.63)	73 (6.8%) 0.65 (0.50, 0.76)	6 (4.8%) 0.67 (0.05, 0.95)	6 (8.3%) 0.83 (0.27, 0.98)
Anti-CD20mab monotherapy → CIT	58 (5.1%) 0.88 (0.74, 0.94)	41 (7.0%) 0.36 (0.20, 0.52)	60 (5.6%) 0.68 (0.53, 0.78)	6 (4.8%) 0.50 (0.06, 0.85)	NR
CIT → CIT	59 (5.2%) 0.76 (0.63, 0.86)	35 (6.0%) 0.51 (0.33, 0.66)	48 (4.5%) 0.64 (0.48, 0.76)	8 (6.3%) 0.75 (0.32, 0.93)	NR
cBTKi monotherapy → BCL2i + Anti-CD20mab only	53 (4.7%) 0.93 (0.79, 0.98)	30 (5.2%) 0.80 (0.57, 0.91)	61 (5.7%) 0.90 (0.78, 0.96)	NR	NR
cBTKi monotherapy → CIT	48 (4.3%) 0.84 (0.70, 0.92)	34 (5.8%) 0.47 (0.28, 0.64)	54 (5.0%) 0.65 (0.50, 0.77)	NR	NR
cBTKi monotherapy → BCL2i mono	47 (4.2%) 0.74 (0.57, 0.85)	26 (4.5%) 0.49 (0.25, 0.69)	40 (3.7%) 0.58 (0.39, 0.74)	NR	NR
CIT → Other	50 (4.4%) 0.64 (0.48, 0.76)	20 (3.4%) 0.44 (0.20, 0.67)	46 (4.3%) 0.62 (0.45, 0.75)	6 (4.8%) 0.67 (0.20, 0.90)	NR
cBTKi monotherapy → cBTKi + Anti-CD20mab only	50 (4.4%) 0.94 (0.78, 0.99)	20 (3.4%) 0.35 (0.10, 0.63)	39 (3.6%) 0.75 (0.54, 0.88)	6 (4.8%) 0.50 (0.06, 0.85)	NR
CIT → BCL2i + Anti-CD20mab only	51 (4.5%) 0.96 (0.85, 0.99)	14 (2.4%) 0.92 (0.54, 0.99)	45 (4.2%) 0.98 (0.84, 1.00)	NR	NR
cBTKi monotherapy → Other	33 (2.9%) 0.70 (0.48, 0.84)	31 (5.3%) 0.39 (0.21, 0.58)	40 (3.7%) 0.62 (0.44, 0.76)	10 (7.9%) 0.25 (0.04, 0.56)	NR
Other → Other	37 (3.3%) 0.73 (0.54, 0.86)	26 (4.5%) 0.51 (0.28, 0.71)	44 (4.1%) 0.63 (0.46, 0.77)	NR	NR
CIT → Anti-CD20mab monotherapy	48 (4.3%) 0.91 (0.78, 0.97)	14 (2.4%) 0.71 (0.34, 0.90)	37 (3.4%) 0.89 (0.72, 0.96)	NR	NR
cBTKi monotherapy → cBTKi + non-BCL2i/non-Anti-CD20mab	35 (3.1%) 0.87 (0.68, 0.95)	18 (3.1%) 0.52 (0.24, 0.75)	33 (3.1%) 0.79 (0.58, 0.90)	7 (5.6%) 0.80 (0.20, 0.97)	NR

* Other race/ethnicity categories with smaller frequency are not reported. NR: not reported as frequency was ≤5. NE: not estimable.

## Data Availability

The data supporting the findings reported in this study have been originated by Flatiron Health, Inc. These de-identified data may be made available upon request and are subject to a license agreement with Flatiron Health; interested researchers should contact <dataaccess@flatiron.com> to determine licensing terms.

## Supplementary Materials

The following supporting information can be downloaded at: https://www.mdpi.com/article/10.3390/cancers17152592/s1, Table S1: Additional rules implemented into the existing business rules for line-of-treatment characterization in the database. Table S2: Classification of treatment regimens into pre-defined categories. Table S3: Baseline patient characteristics of the Main Cohort. Table S4: E-values associated with hazard ratios for each treatment sequences compared in the Analytic Cohort (N = 1711) using Model 1 and Model 2. Figure S1: Kaplan–Meier plot of most frequently observed treatment sequences in the Main Cohort. Figure S2: Durations of line and gap between first two lines within each treatment sequence in the Main Cohort. Figure S3: Adjusted hazard ratios from multivariable Cox proportional hazard models for comparison of OS across most frequently observed treatment sequences in the Main Cohort..

## Abbreviations

The following abbreviations are used in this manuscript:

cBTKi	covalent Bruton tyrosine kinase inhibitors
BCL2i	B-cell lymphoma 2 inhibitors
anti-CD20mab	anti-CD20 monoclonal antibody
CLL/SLL	chronic lymphocytic leukemia/small lymphocytic lymphoma
CIT	ChemoImmunoTherapy (anti-CD20 monoclonal antibody + Chemotherapy)
M1	Model 1
M2	Model 2
