# Supplementary material for: Overall Survival Associated with Real-World Treatment Sequences in Patients with CLL/SLL in the United States
Source: Cancers (Basel). 2025 Aug 7;17(15):2592. doi: 10.3390/cancers17152592 (PMC12346484; doi:10.3390/cancers17152592)
Supplement: Supplementary file 1 [file cancers-17-02592-s001.zip › cancers-3750484-supplementary.pdf]

Supplementary Materials

Suppl. Table S1: Additional rules implemented into the existing business rules for line-of-treatment characterization in the database.

Additional Rules	
1.	For any pair of lines containing these oral anticancer agents, idelalisib, ibrutinib, acalabrutinib, zanubrutinib, lenalidomide, chlorambucil, venetoclax, duvelisib, and pirtobrutinib, if the line name (regimen name) is SAME and the GAP between the two lines is <365 days, then MERGE both lines into one and use minimum start date and maximum end date.
2.	For any pair of lines containing rituximab (or its generics), if the line name (regimen name) is SAME and the GAP between the two lines is <180 days, then MERGE both lines into one and use minimum start date and maximum end date.
3.	For any pair of lines containing ALL other remaining anticancer agents (except those included in rules #1 and #2), if the line name (regimen name) is SAME and the GAP between the two lines is <120 days, then MERGE both lines into one and use minimum start date and maximum end date.
4.	For any pair of lines containing anti-CD20mab monotherapy → anti-CD20mab in combination with another agent, if the duration of anti-CD20mab monotherapy is <21 days AND the GAP between the two lines is ≤30 days, then MERGE the two lines and use minimum start date and maximum end date.
5.	For any pair of lines containing monotherapy with one of these oral anticancer agents, idelalisib, ibrutinib, acalabrutinib, zanubrutinib, lenalidomide, chlorambucil, duvelisib, and pirtobrutinib (i.e., non-venetoclax), followed by the same oral agent in combination with venetoclax, if the duration of oral non-venetoclax monotherapy is <28 days AND the GAP between the two lines is ≤30 days, then MERGE the two lines and use minimum start date and maximum end date.
6.	For any pair of lines containing anti-CD20mab monotherapy → anti-CD20mab in combination with another agent, if the duration of anti-CD20mab monotherapy is <21 days AND the GAP between the two lines is >30 days, then DISREGARD the monotherapy line.
7.	For any pair of lines containing monotherapy with one of these oral anticancer agents, idelalisib, ibrutinib, acalabrutinib, zanubrutinib, lenalidomide, chlorambucil, duvelisib, and pirtobrutinib (i.e., non-venetoclax) followed by the same oral agent in combination with venetoclax, if the duration of oral non-venetoclax monotherapy is <28 days AND the GAP between the two lines is >30 days, then DISREGARD the monotherapy line.

Suppl. Table S2. Classification of treatment regimens into pre-defined categories.

Treatment Category	Treatment Regimen
Investigational	Clinical Study Drug (either alone or in any form of combination)
cBKTi Monotherapy	ibrutinib, acalabrutinib, or zanubrutinib, without any other drugs in the regimen
ncBTKi Monotherapy	Single- agent pirtobrutinib
Anti-CD20mab Monotherapy	rituximab (or other biosimilars), obinutuzumab, or ofatumumab, without any other drugs in the regimen
BCL2i Monotherapy	venetoclax is the only drug in the regimen
BCL2i + Anti-CD20mab Only	venetoclax + rituximab (or other biosimilars), venetoclax + obinutuzumab, or venetoclax + ofatumumab, without any other drugs in the regimen
Chemotherapy Only	Following agents alone or in any combination: bendamustine, busulfan, capecitabine, carboplatin, cisplatin, chlorambucil, cyclophosphamide, cytarabine, docetaxel, doxorubicin, etoposide, fludarabine, fluorouracil, gemcitabine, hydroxyurea, ifosfamide, lenalidomide, methotrexate, mitoxantrone, oxaliplatin, paclitaxel, pemetrexed, prednisone, vincristine (no other drugs beyond this list are present in the regimen)
Anti-CD20mab + Chemotherapy Only (CIT; chemoimmunotherapy)	rituximab (or other biosimilars) AND/OR obinutuzumab AND/OR ofatumumab in combination with chemotherapy agents (see 'chemotherapy only' row above for examples), but without any other drugs in the regimen
BCL2i + cBTKi Only	venetoclax + ibrutinib, venetoclax + acalabrutinib, or venetoclax + zanubrutinib, without any other drugs in the regimen
BCL2i + ncBTKi Only	venetoclax + pirtobrutinib, without any other drugs in the regimen
cBTKi + Anti-CD20mab Only	(One or more of the following: ibrutinib OR acalabrutinib OR zanubrutinib) + (one or more of the following: rituximab (or other biosimilars), OR obinutuzumab, OR ofatumumab), without any other drugs in the regimen
ncBTKi + Anti-CD20mab Only	pirtobrutinib + (one or more of the following: rituximab (or other biosimilars), OR obinutuzumab, OR ofatumumab), without any other drugs in the regimen
BCL2i + cBKTi + Anti-CD20mab Only	venetoclax + (one of the following: ibrutinib, acalabrutinib, or zanubrutinib) + (one or more of the following: rituximab (or other biosimilars), obinutuzumab, or ofatumumab), without any other drugs in the regimen
BCL2i + ncBKTi + Anti-CD20mab Only	venetoclax + pirtobrutinib + (one or more of the following: rituximab (or other biosimilars), obinutuzumab, or ofatumumab), without any other drugs in the regimen
PI3Ki Monotherapy	idelalisib, umbralisib, or duvelisib, without any other drugs in the regimen
PI3Ki + Chemotherapy Only	idelalisib OR umbralisib OR duvelisib in combination with chemotherapy agents (see 'chemotherapy only' row above for examples), but without any other drugs in the regimen
BCL2i + non-BTKi/non-Anti-CD20mab	venetoclax in combination with other agents, however the regimen does not include either a BTKi or Anti-CD20mab
cBTKi + non-BCL2i/non-Anti-CD20mab	ibrutinib OR acalabrutinib OR zanubrutinib in combination with other agents, however the regimen does not include BCL2i or Anti-CD20mab
ncBTKi + non-BCL2i/non-Anti-CD20mab	pirtobrutinib in combination with other agents; however, the regimen cannot include a BCL2i or Anti-CD20mab
Other	All other regimens not categorized above

Suppl. Table S3. Baseline patient characteristics of the Main Cohort.

Characteristics – Main Cohort	Overall<br>N = 2878	Patients Included in Comparative Analyses[a]<br>N = 2235
**Age, median (IQR)**	72 (64, 79)	72 (64, 78)
**Age subgroups, n (%)**		
≤75 years	1853 (64.4)	1435 (64.2)
>75 years	1025 (35.6)	800 (35.8)
**Sex, n (%)**		
Male	1801 (62.6)	1388 (62.1)
Female	1077 (37.4)	847 (37.9)
**Combined Ethnicity and Race, n (%)**		
Non-Hispanic White	1830 (63.6)	1419 (63.5)
Non-Hispanic Black/African American	203 (7.1)	166 (7.4)
Hispanic	126 (4.4)	91 (4.1)
Other[b]	719 (25.0)	559 (25.0)
**Socioeconomic status[c], n (%)**		
1	344 (12.0)	270 (12.1)
2	445 (15.5)	346 (15.5)
3	548 (19.0)	429 (19.2)
4	679 (23.6)	542 (24.3)
5	655 (22.8)	494 (22.1)
Missing	207 (7.2)	154 (6.9)
**Practice type, n (%)**		
Academic	405 (14.1)	312 (14.0)
Community	2473 (85.9)	1923 (86.0)
**Disease subtype, n (%)**		
CLL	2644 (91.9)	2046 (91.5)
SLL	234 (8.1)	189 (8.5)
**Year of initiation of first LoT, n (%)**		
2016	570 (19.8)	463 (20.7)
2017	500 (17.4)	421 (18.8)
2018	445 (15.5)	351 (15.7)
2019	429 (14.9)	322 (14.4)
2020	349 (12.1)	256 (11.5)
2021	311 (10.8)	217 (9.7)
2022	203 (7.1)	150 (6.7)
2023	71 (2.5)	55 (2.5)

**Total LoTs received, n (%)**		
2	1898 (65.9)	1512 (67.7)
3	623 (21.6)	464 (20.8)
4+	357 (12.4)	259 (11.6)
**Patients with data available[d]**		
**ECOG PS, n (%)**	**n = 2036**	**n = 1590**
0-1	1869 (91.8)	1454 (91.4)
2-4	167 (8.2)	136 (8.6)
**Rai Stage, n (%)**	**n = 1716**	**n = 1345**
0-I	1115 (65.0)	862 (64.1)
II-IV	601 (35.0)	483 (35.9)
**IGHV, n (%)**	**n = 1 074**	**n = 843**
Mutated	415 (38.6)	323 (38.3)
Unmutated	659 (61.4)	520 (61.7)
**del(11q)**	**n = 2233**	**n = 1752**
No	1838 (82.3)	1448 (82.6)
Yes	395 (17.7)	304 (17.4)
**del(13q)**	**n = 2249**	**n = 1768**
No	1223 (54.4)	957 (54.1)
Yes	1026 (45.6)	811 (45.9)
**del(17p)/TP53**	**n = 2249**	**n = 1766**
No	1981 (88.1)	1567 (88.7)
Yes	268 (11.9)	199 (11.3)
**Trisomy 12**	**n = 2223**	**n = 1749**
No	1629 (73.3)	1282 (73.3)
Yes	594 (26.7)	467 (26.7)

[a]Included patients who received the most common treatment sequences (n ≥ 50)
[b]Other includes 27 (0.9%) Asian, 82 (2.8%) other, and 610 (21.2%) missing/unknown in the overall population; and 21 (0.9%) Asian, 66 (3.0%) other, and 472 (21.1%) missing/unknown in the comparative analysis population.
[c]Socioeconomic status: an area-level indicator of patients' socioeconomic status *(1-low; 5-high)*, which was calculated according to the Yost Index (incorporating income, home values, rental costs, poverty, blue-collar employment, unemployment, and education information) using Census block group (ie, neighborhood) data from the American Community Survey (2015-2019). [1,2]
[d]Excludes patients with missing data.

[1] Yost, K.; Perkins, C.; Cohen, R.; Morris, C.; Wright, W. Socioeconomic status and breast cancer incidence in California for different race/ethnic groups. *Cancer Causes Control* **2001**, *12*, 703-711, doi:10.1023/a:1011240019516.
[2] Guadamuz, J.S.; Wang, X.; Ryals, C.A.; Miksad, R.A.; Snider, J.; Walters, J.; Calip, G.S. Socioeconomic status and inequities in treatment initiation and survival among patients with cancer, 2011-2022. *JNCI Cancer Spectrum* **2023**, *7*, doi:10.1093/jncics/pkad058.

Suppl. Table S4 E-values associated with hazard ratios for each treatment sequences compared in the Analytic Cohort (N = 1711) using Model 1 and Model 2.

	Model 1		Model 2	
Treatment Sequence vs. reference*	e-values	HR (95% CI)	e-values	HR (95% CI)
CIT → cBTKi monotherapy	1.778	1.36 (0.76-2.44)	1.70	1.31 (0.73-2.35)
cBTKi monotherapy → CIT	2.729	2.11 (1.08-4.09)	1.984	1.50 (0.77-2.93)
cBTKi monotherapy → BCL2i monotherapy	2.398	1.83 (0.92-3.61)	2.175	1.65 (0.83-3.26)
CIT → Other	4.713	4.18 (2.23-7.85)	3.309	2.65 (1.38-5.07)
cBTKi monotherapy → cBTKi + Anti-CD20mab	2.314	1.76 (0.80-3.85)	1.795	1.37 (0.63-3.01)
CIT → BCL2i + Anti-CD20mab	1.520	0.84 (0.36-1.96)	1.515	0.84 (0.36-1.97)
cBTKi monotherapy → Other	3.195	2.54 (1.30-4.95)	2.491	1.90 (0.97-3.72)
Other → Other	3.211	2.55 (1.30-5.00)	1.306	1.09 (0.54-2.17)
CIT → Anti-CD20mab monotherapy	1.761	1.35 (0.64-2.84)	1.185	1.04 (0.49-2.19)
cBTKi monotherapy → cBTKi+non-BCL2/non-anti-CD20mab	2.332	1.77 (0.83-3.78)	1.861	1.42 (0.66-3.03)
Anti-CD20mab monotherapy → cBTKi monotherapy	2.437	1.86 (1.03-3.36)	1.864	1.42 (0.78-2.58)
Anti-CD20mab monotherapy → BCL2i + Anti-CD20mab	1.732	1.33 (0.66-2.67)	1.357	1.11 (0.55-2.24)
cBTKi monotherapy → Anti-CD20mab monotherapy	3.130	2.47 (1.32-4.63)	2.602	2.00 (1.07-3.74)
Anti-CD20mab monotherapy → CIT	3.347	2.68 (1.42-5.07)	2.552	1.95 (1.03-3.69)
CIT → CIT	3.529	2.87 (1.54-5.34)	2.935	2.29 (1.23-4.28)

*Reference sequence: cBTKi monotherapy → BCL2i + Anti-CD20 mab

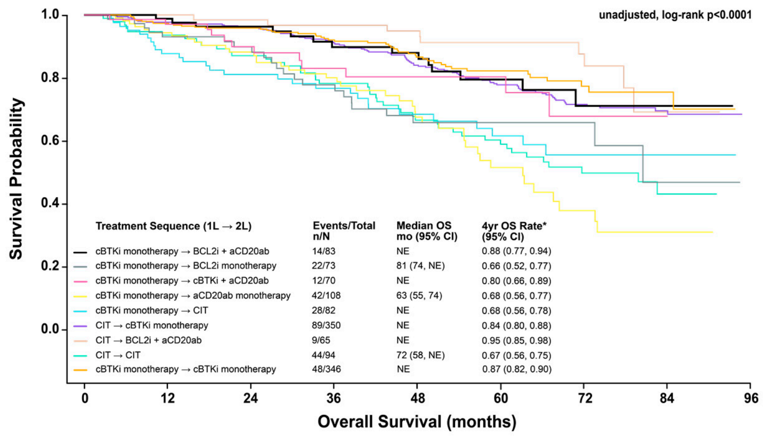

Numbers at Risk:

Months	0	12	24	36	48	60	72	84	96
	83	79	70	52	46	26	14	7	0
	73	65	53	41	29	18	10	3	0
	70	65	47	31	24	16	6	1	0
	108	96	80	63	39	20	13	5	0
	82	69	57	49	35	25	11	5	0
	350	336	319	296	255	211	145	68	0
	65	63	59	55	52	40	23	7	0
	94	86	80	70	55	45	26	10	0
	346	325	286	224	159	99	44	16	0

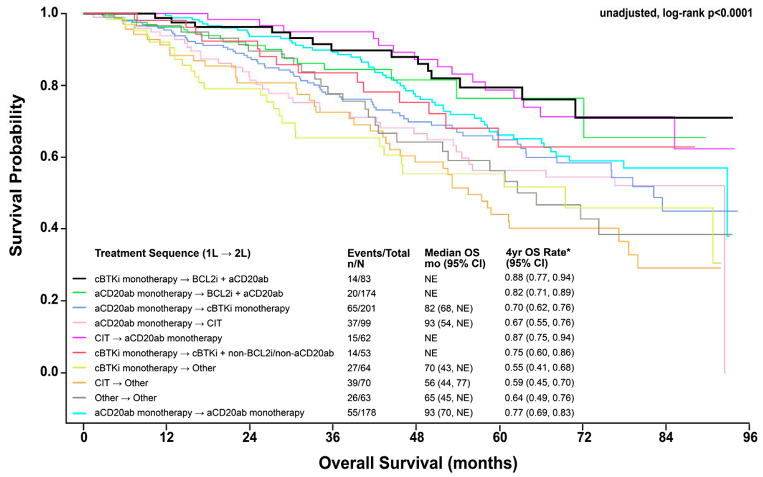

Numbers at Risk:

Months	0	12	24	36	48	60	72	84	96
	83	79	70	52	46	26	14	7	0
	174	132	88	46	23	13	7	2	0
	201	182	154	117	80	58	32	11	0
	99	89	69	52	41	32	25	15	0
	62	62	58	51	44	34	23	10	0
	53	51	43	35	26	12	10	2	0
	64	57	43	29	21	15	8	4	0
	70	62	51	43	34	24	15	6	0
	63	56	50	38	26	18	11	7	0
	178	176	160	138	100	66	44	22	0

*Point estimate for OS at 4 years (48mo) based on survival distribution function

Other included any treatment regimen that was not classified as cBTKi monotherapy, non-cBTKi monotherapy, aCD20ab monotherapy, BCL2i monotherapy, PI3Ki monotherapy, BCL2i + aCD20ab only, chemotherapy only, chemoimmunotherapy (aCD20ab + chemotherapy only), BCL2i + cBTKi only, BCL2i + ncBTKi only, cBTKi + aCD20ab only, ncBTKi + aCD20ab only, BCL2i + cBKTi + aCD20ab only, BCL2i + ncBKTi + aCD20ab only, PI3Ki + chemotherapy only, BCL2i + non-BTKi/non-aCD20ab, cBTKi + non-BCL2i/non-aCD20ab, and ncBTKi + non-BCL2i/non-aCD20ab.

CIT: chemoimmunotherapy (anti-CD20 monoclonal antibody + chemotherapy).

Note: KM plot was split for visualization purposes only.

Suppl. Figure S1. Kaplan–Meier plot of most frequently observed treatment sequences in the Main Cohort.

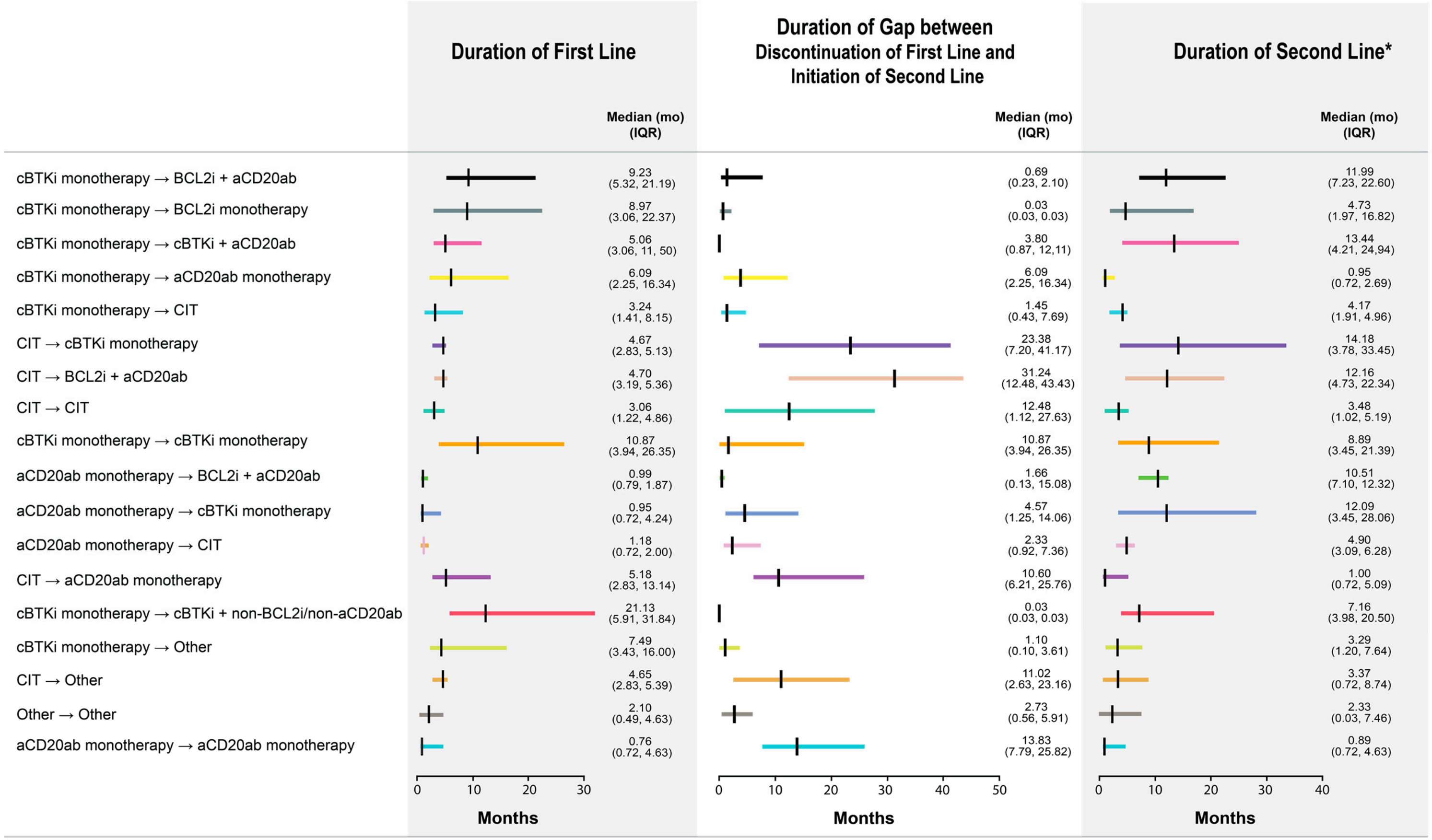

Vertical bars represent median duration of each line or gap between two lines within a sequence.
Horizontal bars represent interquartile range around the median duration of each line or gap between two lines within a sequence.
*These durations are not accounted for censoring as not all patients received a subsequent line of treatment and are descriptive only.

Suppl. Figure S2. Durations of line and gap between first two lines within each treatment sequence in the Main Cohort.

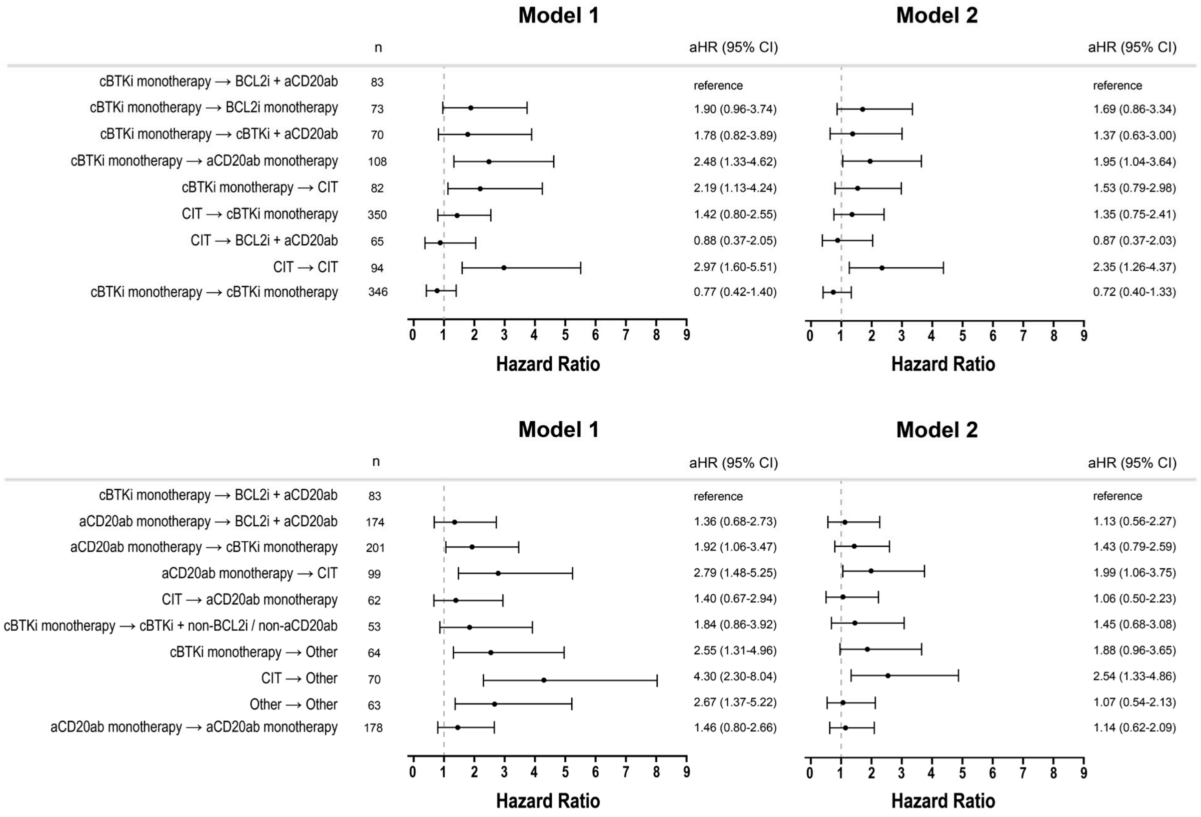

Model 1 adjusted for baseline (at/prior to index date) factors including age at index, sex, race/ethnicity, socioeconomic status, practice type, disease subtype, Rai stage, ECOG at index, d11q, d13q, tri12, del17p/TP53, IGHV, index year, and time since initial diagnosis to index.
Model 2 additionally adjusted for cumulative number of lines of therapy received along with its duration as a blended time-dependent covariate to account for potential differences in OS due to treatments received beyond first two lines of therapy.
Reference sequence: cBTKi monotherapy → BCL2i + anti-CD20 monoclonal antibody.
CIT: chemoimmunotherapy (anti-CD20 monoclonal antibody + chemotherapy).
Note: Forest plot was split for visualization purposes only.

Suppl. Figure S3. Adjusted hazard ratios from multivariable Cox proportional hazard models for comparison of os across most frequently observed treatment sequences in the Main Cohort.
